# On a Successful Mode of Treating Puerperal Fever

**Published:** 1816-07

**Authors:** S. Shath

**Affiliations:** of Dunstone, near Kingsbridge, Devon.


THE LONDON
### Medical and Physical Journal.

1 OF VOL. XXXVI.] JULY, 1816. [NO. 209.

"For many fortunate discoveries in medicine, and for the detection of nume-
"rous errors, the world is indebted to the rapid circulation of Monthly
"Journals; and there never existed any work to which the Faculty in
"EUROPE and AMERICA were under deeper obligations than to the
"Medical and Physical Journal of London, now forming a long, but an
"invaluable, series."—RUSH.

*For the London Medical and Physical Journal.*

### *On a successful Mode of Treating Puerperal Fever;* by Dr. S. SHATH, of Dunstone, near Kingsbridge, Devon.

HAVING followed a mode of practice in the Puerperal Fever, for more than forty years past, with the greatest possible success, and conceiving it to be a plan not universally known, I think that it will be found worthy of a place in your most useful and extensive publication; and I trust that every practitioner will find it equally successful, if the modus operandi is strictly attended to.

As I am writing to gentlemen of the profession, I need not say any thing of the nature of the disorder or its symptoms, but shall immediately proceed to the method, in as clear and concise a manner as possible.

When called to a patient labouring under puerperal fever, or in childbed, with the abdomen tense and sore, I immediately pass into the uterus *(mea manu)* an injection composed of eight ounces of equal parts of warm milk and water, with half an ounce of moist sugar dissolved in it; and soon after let a pint of warm milk and water, with an ounce of sugar and an ounce of butter or oil, be injected into the rectum; both of which should be repeated four or five times in twenty-four hours, unless the complaints entirely subside. The abdomen is also fomented with warm water, and then the following liniment rubbed in all over the abdomen and labia pudendi, three or four times in twenty-four hours.

℞. Digitalis pulv. ʒj

Tinct. Digital. ʒij.

Ol. Oliv. Opt. ℥ſs.

Adipis Suillæ ʒvi. fiat Linimentum.

To inject the uterus, you should be provided with a tube of the same size and perforated in the same manner as a common catheter, but not less than seven or eight inches in length, length. I have twice found the uterus situated so high as to require a longer tube, for which purpose I have used a male catheter straitened. The tube being introduced in the uterus, and your injection put into a bladder with a small pipe fixed to it, properly adjusted to the tube, you join them together, and gently press it into the uterus, having a sponge at the os externum to receive it as it returns, in order to avoid wetting the bed.

With regard to medicines, I have always found it necessary to give calomel and pulvis antimonialis in small doses, frequently repeated, and the saline draughts, with the addition of the tinct. digitalis, which contributes greatly to its effect in abating fever; and when the patient is in much pain, or distressed for want of sleep, I give the tincture or extract of hyocyamus, which I find in general preferable to opium.

I was led to this practice by a case mentioned in Laz. Riverius, p. 486, chap. 24, de Morbis Acutis Puerperarum. Fracifurt, ann. 1669.

"Contingit etiam in nonnullis orificium uteri statim a partu adeo constringi, ut detentus intra uterum sanguis subito grumescens, et putrefactus sævissima inducat symptomata, cumque nulla arte exitus ei parari possit, præsentaneam mortem inferat. Refert tamen Harvæus loco citato historiam mulieris a se curatæ cum tali affectu laboraret. Pudendi labra tumida erant, et fervida; orificium uteri durum, et clausum, ipse vero instrumento ferreo immisso per vim aliqantulum aperuit ut injectionem per siphunculum admitteret; indeque grumescens, ater fœtidusque sanguis ad libras aliquot effluxit, cum presenti ægræ levamine."

It was some years after I had begun to inject the uterus and rectum that Mauriceau's Midwifery, printed Paris 1715, fell into my hands, and there I see that he advises the same method. There are cases mentioned in the 2d vol. which fully confirm its utility; and, if it doth not trespass too much on your pages, should thank you for inserting the following extract:

"On luy donnera ausi des clysteres qui puissent attirer les humeurs en bas; et on lui etuvera les parties basses d'une decoction émolliente et aperitive, faite avec les mauves, parietaire, camomille, melilot, racines d'asperges, et la graine de lin; de laquelle decoction on pourra aussi faire injection dans la matrice." Vol. i. p. 418.

*June* 8, 1816.

---

We feel highly obliged to so experienced and respectable a practitioner for the above article; and shall be further thankful for any pointed histories which his case-book may furnish.—Edit.

